# Cell‐extracellular matrix interactions in the fluidic phase direct the topology and polarity of self‐organized epithelial structures

**DOI:** 10.1111/cpr.13014

**Published:** 2021-02-21

**Authors:** Mingxing Ouyang, Jiun‐Yann Yu, Yenyu Chen, Linhong Deng, Chin‐Lin Guo

**Affiliations:** ^1^ Institute of Biomedical Engineering and Health Sciences School of Pharmacy & School of Medicine Changzhou University Changzhou China; ^2^ Department of Bioengineering California Institute of Technology Pasadena USA

**Keywords:** cell‐ECM interaction, epithelial polarity, epithelial self‐assembly, epithelial topology, fluidic phase, tubulogenesis

## Abstract

**Introduction:**

In vivo, cells are surrounded by extracellular matrix (ECM). To build organs from single cells, it is generally believed that ECM serves as scaffolds to coordinate cell positioning and differentiation. Nevertheless, how cells utilize cell‐ECM interactions for the spatiotemporal coordination to different ECM at the tissue scale is not fully understood.

**Methods:**

Here, using in vitro assay with engineered MDCK cells expressing H2B‐mCherry (nucleus) and gp135/Podocalyxin‐GFP (apical marker), we show in multi‐dimensions that such coordination for epithelial morphogenesis can be determined by cell‐soluble ECM interaction in the fluidic phase.

**Results:**

The coordination depends on the native topology of ECM components such as sheet‐like basement membrane (BM) and type I collagen (COL) fibres: scaffold formed by BM (COL) facilitates a close‐ended (open‐ended) coordination that leads to the formation of lobular (tubular) epithelium. Further, cells form apicobasal polarity throughout the entire lobule/tubule without a complete coverage of ECM at the basal side, and time‐lapse two‐photon scanning imaging reveals the polarization occurring early and maintained through the lobular expansion. During polarization, gp135‐GFP was converged to the apical surface collectively in the lobular/tubular structures, suggesting possible intercellular communications. Under suspension culture, the polarization was impaired with multi‐lumen formation in the tubules, implying the importance of ECM biomechanical microenvironment.

**Conclusion:**

Our results suggest a biophysical mechanism for cells to form polarity and coordinate positioning at tissue scale, and in engineering epithelium through cell‐soluble ECM interaction and self‐assembly.

## Graphical abstract

MDCK cells are cultured on the top of basement membrane (BM) gel with different soluble ECM components (BM or COL) in the medium. Cells grow into lobular (with soluble BM) or tubular (with soluble COL) epithelial structures with apicobasal polarity (on the right). Cells express H2B‐mCherry and gp135‐GFP to indicate their nucleus and polarity, respectively.

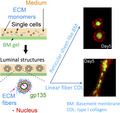

## 1 INTRODUCTION

One feature in epithelial development and regeneration is the spatiotemporal coordination of cell positioning and differentiation.^1^ In these processes, cells develop apicobasal polarity and form lobular or tubular sheets.^2^, ^3^ Most remarkably, they can coordinate the orientation of polarity throughout the entire tissue.^3^ Loss of such coordination is often a hallmark of tumours.^4^ As such, understanding how epithelial cells coordinate their positioning and polarization is not only essential for developmental biology and regenerative medicine, but also important for cancer biology. The transmembrane glycoprotein gp135 (also called Podocalyxin) is often used as an apical marker located at the luminal surfaces,^5^, ^6^ and Rab small GTPases mediate the direct transportation of gp135 and apicobasal polarization.^7^, ^8^ Over the past few decades, studies on epithelial morphogenesis have indicated the importance of cell‐cell adhesions and cell‐extracellular matrix (ECM) interactions.^5^, ^9^, ^10^, ^11^, ^12^, ^13^, ^14^, ^15^, ^16^ The differentiation of epithelial cells in which non‐polarized cells form polarized epithelium depends on ECM components.^9^ It was shown that breast epithelial cells differentiate into tubules in type I collagen (COL),^17^, ^18^ while they form lobular acini in basement membrane (BM, mimicked by Matrigel in experiments).^19^, ^20^ Our previous study further showed that cell‐collagen interaction permits a long‐range morphogenetic coordination at sub‐millimetre scale.^21^ Nevertheless, how epithelial cells utilize cell‐ECM interaction to coordinate their positioning and polarization in response to different ECM components at the whole‐tissue scale is not fully understood.

To form long‐range coordination, it is generally believed that ECM can serve as scaffolds to guide cell positioning and polarization.^15^ Cells constantly secrete soluble ECM molecules and degrade existing ECM scaffolds into soluble fragments in vivo. Theoretically, these soluble forms of ECM can be assembled into scaffolds through two processes. The first is that they self‐assemble into new scaffolds or re‐incorporate into pre‐existing scaffolds. Alternatively, soluble ECM can interact with cells which serve as nucleation cores to assemble ECM scaffolds. An example is the development of renal tubules where BM components are found to be dynamically assembled around the pre‐tubular structure.^22^ Apicobasal polarization is a typical process during epithelial tubulogenesis, and consistently with the role from ECM, the cellular mechanism involving integrin and RhoA signalling pathways has been identified in triggering the polarity formation.^23^, ^24^

Here, we study whether ECM scaffolds created by ECM self‐assembled hydrogel or by cell‐mediated assembly play the primary roles in the formation and coordination of epithelial morphogenesis. These two processes can hardly be decoupled in vivo or through the conventional ECM reconstitution approaches. Our recent work demonstrated that cell motion promotes fibrillary assembly of soluble COL, in supporting the role of cells in ECM scaffold generation.^25^ We therefore use the in vitro open‐system assay, and found that spatiotemporal coordination in epithelial morphogenesis and polarization can occur on cell‐assembled ECM in the fluidic phase rather than pre‐assembled ECM in the solid phase. The coordination depends on native topology of the ECM components such as basement membrane (BM) and type I collagen (COL). Further discovered during tubulogenesis, the apicobasal polarization proceeds in a collective way along the axis of the tubule, implying intercellular communications within the cell groups. Our results suggest a potential mechanism which cells can use to form polarity and coordinate morphogenesis in vivo, and a strategy to engineer epithelial structures through self‐assembly in vitro.

## 2 MATERIALS AND METHODS

### 2.1 Cell Culture, reagents, DNA constructs and lentivirus

Cell culture medium and reagents were purchased from Invitrogen Gibco. Madin Darby canine kidney (MDCK II) cells (from ATCC) were maintained in Advanced Dulbecco's modified Eagle's medium (serum reduced medium) supplemented with 3% fetal bovine serum, 2 mM L‐glutamine, 20 unit/mL penicillin, 20 μg/mL streptomycin and 1 mM sodium pyruvate in a humidified 95% air, 5% CO_2_ incubator at 37°C.

BD Matrigel (basement membrane matrix, growth factor reduced and phenol red‐free) and 3‐D Culture Matrix™ Rat Collagen I (5 mg/mL) were purchased from BD Biosciences and R&D Systems, respectively. Rabbit anti‐laminin and mouse anti‐collagen I primary antibodies were purchased from Sigma, and mouse anti‐integrin ɑ6 antibody from Abcam. Pacific Blue‐conjugated goat anti‐rabbit and anti‐mouse IgGs were purchased from Invitrogen, and Rhodamine‐conjugated goat anti‐mouse IgG antibody from Sigma.

The plasmid pcDNA3‐gp135‐GFP construct was a gift from Dr Joachim Füllekrug (Max Planck Institute of Molecular Cell Biology and Genetics).^26^ The plasmid expressing GFP‐tagged human β‐actin (GFP‐β‐actin) under endogenous promoter was provided by Dr Beat A. Imhof (Switzerland).^27^ Lentivirus encoding mCherry‐conjugated histone H2B (H2B‐mCherry) was generously provided by Dr Rusty Lansford and David Huss (Biology, California Institute of Technology).^28^

### 2.2 Development of stable fluorescent MDCK cell lines

We first developed the stable MDCK cell line expressing H2B‐mCherry. Cells at 20%‐30% confluency were infected with lentivirus encoding H2B‐mCherry and then diluted in 96‐well plates to enable the selection and the expansion of single fluorescent colony. Next, we transfected MDCK_H2B‐mCherry cells with pcDNA3‐gp135‐EGFP to develop the cell line expressing both H2B‐mCherry and gp135‐EGFP (MDCK_H2B‐mCherry/gp135‐EGFP). In addition, we have transfected MDCK cells with EGFP‐β‐actin plasmid (MDCK_EGFP‐actin). This cell line was used to compare the results obtained from MDCK_H2B‐mCherry/gp135‐EGFP cells. The transfection was performed by using Lipofectamine2000 (Invitrogen), followed by the antibiotic selection with G418 (300 µg/mL) for 2 weeks to obtain a cell pool displaying various levels of EGFP. A single colony with an intermediate fluorescence intensity of gp135‐GFP or GFP‐β‐actin was selected through dilutions of the cell pool in 96‐well plates and further amplified.

### 2.3 Chambers for cell culture and microscopy

Cell culture experiments and time‐lapse microscopy were performed in custom, stainless steel chambers. These chambers were manufactured to have a rectangular shape with a height of 0.6 cm and a 2 × 5.5 cm^2^ internal opening. Nail polish was used to seal 24 × 60 mm No. 1 coverslips on the bottom of the chambers. To perform multiple‐well experiments in one chamber, polydimethylsiloxane (PDMS) blocks were cut to fit the chamber containing multiple wells (~0.5 × 0.5 cm^2^). The surface of PDMS block was then cleaned and air‐dried to allow for a firm attachment on the coverslip of the chamber.

### 2.4 Cell culture on BM gels or less‐adhesive substrates

To make BM gels, we prepared sterile chambers sealed with coverslips on the bottom, and the stock solution (100%) of BD Matrigel was then spread on the top of the coverslips (40‐80 µL/cm^2^) followed by incubation at 37°C for 20‐30 minutes. This allowed forming a layer of gel with a variable height (200‐400 µm).

To place cells, individual MDCK cells (~1‐2 × 10^4^ cells/cm^2^) were seeded on BM gels in the culture medium containing 2% BM or 20 µg/mL COL. Then, the chamber was placed into petri dish in the cell culture incubator with medium change every 4 days, or every day (or every other day) during time‐lapse microscopy. Here, the concentration of BM in the medium followed the ‘on‐top’ assay developed by Bissell and her coworkers.^29^ 20 µg/mL COL in the medium was chosen to match the mass concentration of 2% BM (10 mg/mL in stock). Nevertheless, we have applied the different concentrations of COL from 5 to 50 µg/mL, and cells could form tubular structures within this range.

For cell culture on less‐adhesive substrates, we first prepared a layer of agarose gel (1%) on the coverslips in the chambers. A mixture of MDCK cells (~10^4^ cells/mL) and culture medium containing 2% BM or 20 µg/mL COL was added into the chambers and moved in the cell culture incubator. To minimize water evaporation, the chambers were covered by coverslips with a small opening (~5 mm) to allow for air exchange. Medium was changed carefully after 7 days to avoid destroying or losing the cell aggregates.

### 2.5 Immuno‐staining experiments

Immuno‐staining experiments were conducted at room temperature, except for incubation with primary antibodies at 4°C. In brief, cell samples were fixed with 4% paraformaldehyde for 15 minutes and permeabilized with 0.1% Triton X‐100 for 20 minutes. The cells were then incubated with rabbit anti‐laminin, or mouse anti‐collagen I at 4°C overnight, followed by incubation with goat secondary antibody conjugated with Pacific Blue (410/455 nm) or Rhodamine (550/570 nm) for 2 hours at room temperature. The images were collected by using epifluorescence or scanning microscopy.

### 2.6 Epifluorescence and scanning microscopy

Olympus IX71 was equipped with automatic XYZ stage (MS‐2000, ASI) and piezo‐electric objective stage (P‐721 Pifoc, Physik Instrumente) for fast multi‐position, z‐scanning and auto‐focusing time‐lapse imaging. An environmental chamber (Haison) was used to maintain humidity, CO_2_ concentration (5%) and temperature (37°C). For phase‐contrast and/or epi‐fluorescence microscopy, the imaging system based on Olympus IX71 microscope was equipped with motorized excitation and emission filters with a shutter control (lambda 10‐3, Sutter), an Electron‐Multiplying CCD camera (ImagEM, C9100‐13, Hamamatsu, 512 × 512 pixels, cooled down to −95°C by water circulation) and a 120W fluorescent illumination lamp (X‐CITE 120Q, EXFO, Lumen Dynamics Group Inc). For confocal scanning microscopy, the Olympus IX71 microscope was equipped with lasers of three wavelengths (405 nm, 475 nm and 594 nm), photomultiplier tubes (H10425 and H7422‐40, Hamamatsu). The two‐photon scanning microscope set up on Olympus IX71 was equipped with a Mai‐Tai^TM^ femtosecond laser source (Spectra‐Physics). 20x objective (NA: 0.45, Olympus) and 10x objective (NA: 0.3, Olympus) were used in the experiments.

### 2.7 Image acquisition and analysis

For phase‐contrast and/or epifluorescence time‐lapse microscopy, Metamorph (version 7.7.3) was used to control the devices and the image acquisition. To acquire z‐stack, epifluorescent images were taken at 21 planes with a step size of 4.5 µm. 3‐D projection was obtained by collecting pixels with the maximal intensity through the entire z‐stack into a single plane by using Metamorph or Matlab programs. For scanning microscopy, Labview was used to run automatic scanning and image acquisition. The exposure time and gains were adjusted according to the image quality and the photo‐bleaching effect. Metamorph, Labview and ImageJ were used for image analysis.

## 3 RESULTS

### 3.1 Cell‐ECM interactions in the fluidic phase for polarized epithelium formation

**FIGURE 1:**
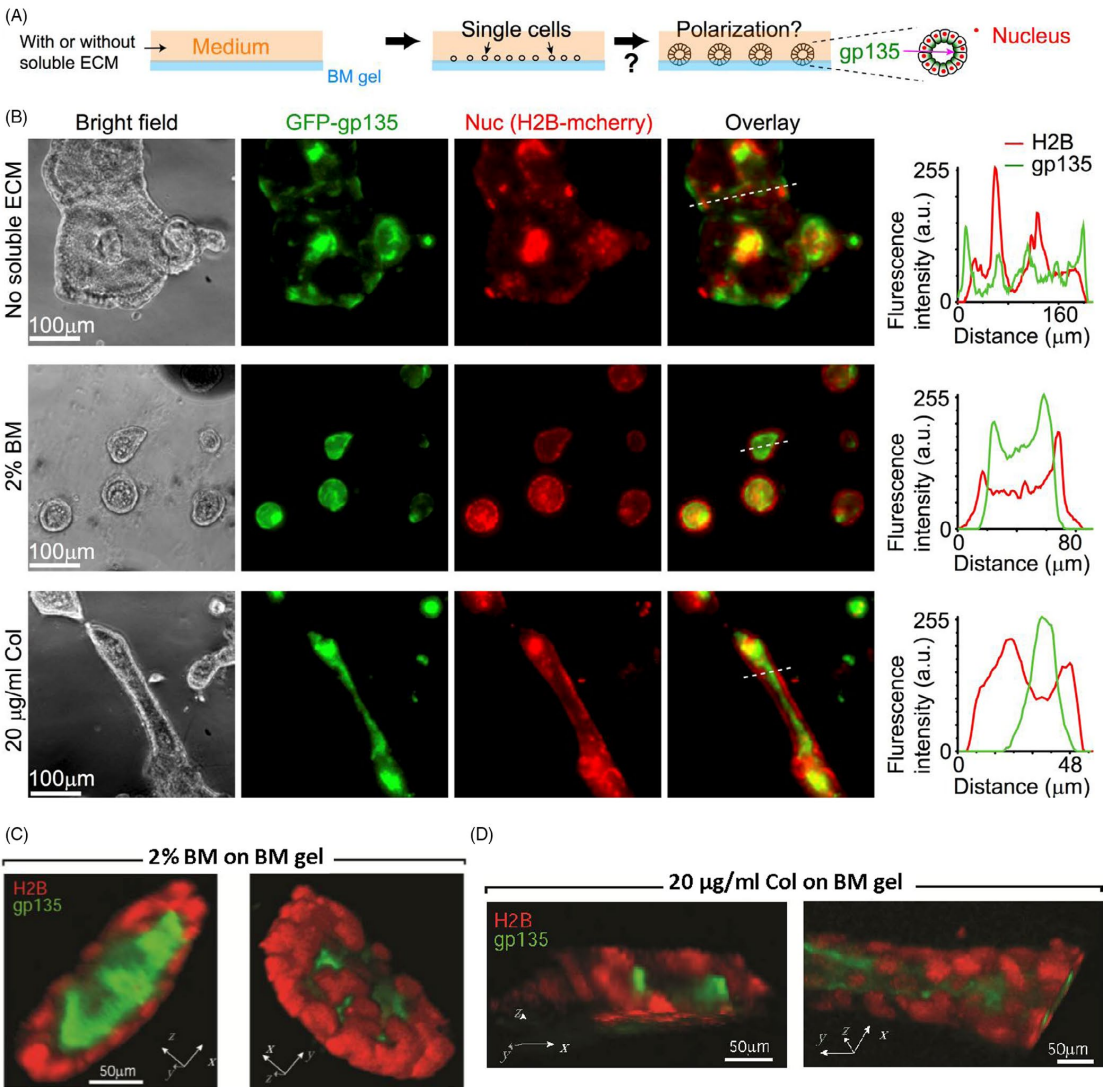
Soluble ECM components are required to form epithelial polarization on Matrigel. (A) Experimental set‐up. MDCK cells were cultured on the top of basement membrane (BM) gels with or without soluble ECM components in the medium. Cells express H2B‐mCherry and gp135‐GFP to indicate their nucleus and polarity, respectively. Note the distribution of nucleus with respect to gp135 in polarized lumens (on the right). (B) Left in each row: Represented phase‐contrast (bright field) and 3‐D projected fluorescent images (gp135: green, H2B: red, and their overlay) for cells seeded on BM gels and cultured for 12 days (seen in Methods for 3‐D projected imaging and processing). The medium contained (top row) no soluble ECM, (middle row) 2% BM or (bottom row) 20 µg/mL type I collagen (COL). Nuc: cell nucleus. Right in each row: Fluorescence intensities of H2B‐mCherry and gp135‐GFP along the indicated white dotted lines (from left to right). The overlay of the red and green curves indicates the positions of cell nucleus (red) with respect to the apical marker (green). a. u.: arbitrary unit. (C and D) 3‐D view of the polarized lobular and tubular structures. The images from confocal scanning microscopy (with 20x objective) were reconstructed into 3‐D structures. The 3‐D views showed the relative position of cell nucleus (red) and apical marker gp135 (green) in the closed lobular (C) and elongated tubular (D) structures

We first examined if cells can develop coordinated polarity on ECM scaffolds formed by ECM self‐assembly without any soluble ECM. Madin‐Darby Canine Kidney (MDCK) cells were used in this study, which are a popular model cell line for epithelial morphogenesis and apicobasal polarity formation regulated by Rab GTPases.^3^, ^8^ To track the development of apicobasal polarity, we engineered MDCK cells stably expressing mCherry‐conjugated histone H2B (H2B‐mCherry) and GFP‐conjugated gp135 (gp135‐GFP). Here, H2B‐mCherry is used to indicate cell nucleus,^28^ whereas the apical marker gp135 is used to indicate cell polarization.^5^, ^26^ To define polarity, we noted that in polarized epithelium, cells are organized into a surrounding and continuous monolayer structure with gp135 primarily confined at their apical surfaces,^5^ which are located at the inner space of the organization (illustrated in Figure 1A with the experimental data shown in Figure 1B). Thus, we used the spatial distribution of H2B‐mCherry with respect to gp135‐GFP to define and track the formation of polarity. The polarization process here refers to the convergence of diffusive gp135‐GFP to the apical surface of the lumen or to the intercellular region between cell nuclei. To initiate cell density‐dependent long‐range coordination, we adopted the cell density characterized and optimized in our previous study.^21^

After long‐term culture, epithelial cells including MDCK cells can secrete ECM molecules.^16^, ^30^, ^31^ We therefore used open systems to dilute and/or remove secreted ECM by changing medium every day or every other day. Cells were cultured on pre‐assembled BD Matrigel gels (to mimicking basement membrane (BM), the primary matrix components underlying polarized epithelium in vivo) open to a large medium space that contained no soluble ECM (Figure 1A). Under this condition, cells grew and merged into big clusters (hundreds of micrometres in diameter) without forming general apicobasal polarity (Figure 1B, top row). In order to visualize the cultured epithelium, 3‐D projection of the epifluorescence images taken at 21 planes with a step size of 4.5 µm was obtained by collecting pixels with the maximal intensity through the entire z‐stack into a single plane, which is further explained in the Methods.

We then examined if cell‐ECM interactions in the fluidic phase are required for polarity formation. Two types of medium were prepared: medium with BM (2%, diluted Matrigel solution from ~ 10 mg/mL stock concentration) and medium with COL (20 µg/mL). From the manufacturer, the major components of Matrigel are laminin (~61%), collagen IV (~30%) and Entactin (~7%). The BM concentration was adapted from studies by our group and others,^7^, ^9^, ^19^, ^21^, ^23^ and the COL concentration at 20 µg/mL was designed to match the similar mass concentration of 2% BM. Here, we used low concentrations of soluble ECM components to prohibit their fast self‐assembly into hydrogel‐like. This allowed cells to live in a fluidic or semi‐fluidic condition and interact with soluble ECM during their proliferation. Here semi‐fluidic condition referred to cells/clusters culturing on solid Matrigel scaffold with direct exposure to the medium. In response to soluble BM, cells were found to form spherical, lobular structures with coordinated polarity (Figure 1B, middle row), whereas for soluble COL, they formed polarized, tubular structures (Figure 1B, bottom row). In both cases, gp135 was confined within the apical area (Figure 1B, middle and bottom rows, right). 3‐D view of these cultured lobular and tubular structures from confocal scanning microscopy further confirmed that cells grew into fine 3‐D structures with gp135 confined at the apical surfaces (Figure 1C,D).

### 3.2 Dynamics of cell positioning and polarization dependent on cell‐ECM interactions

Having shown that cell‐BM (COL) interactions in the fluidic phase lead to the formation of polarized lobules (tubules), we examined how cells coordinate their positioning and polarization in response to soluble BM (COL). Same as the set‐ups in Figure 1A, cells were seeded on 3‐D BM with or without soluble ECM in the medium, and the dynamic processes of cell aggregation and polarization were continuously recorded by time‐lapse imaging in the following days.

**FIGURE 2:**
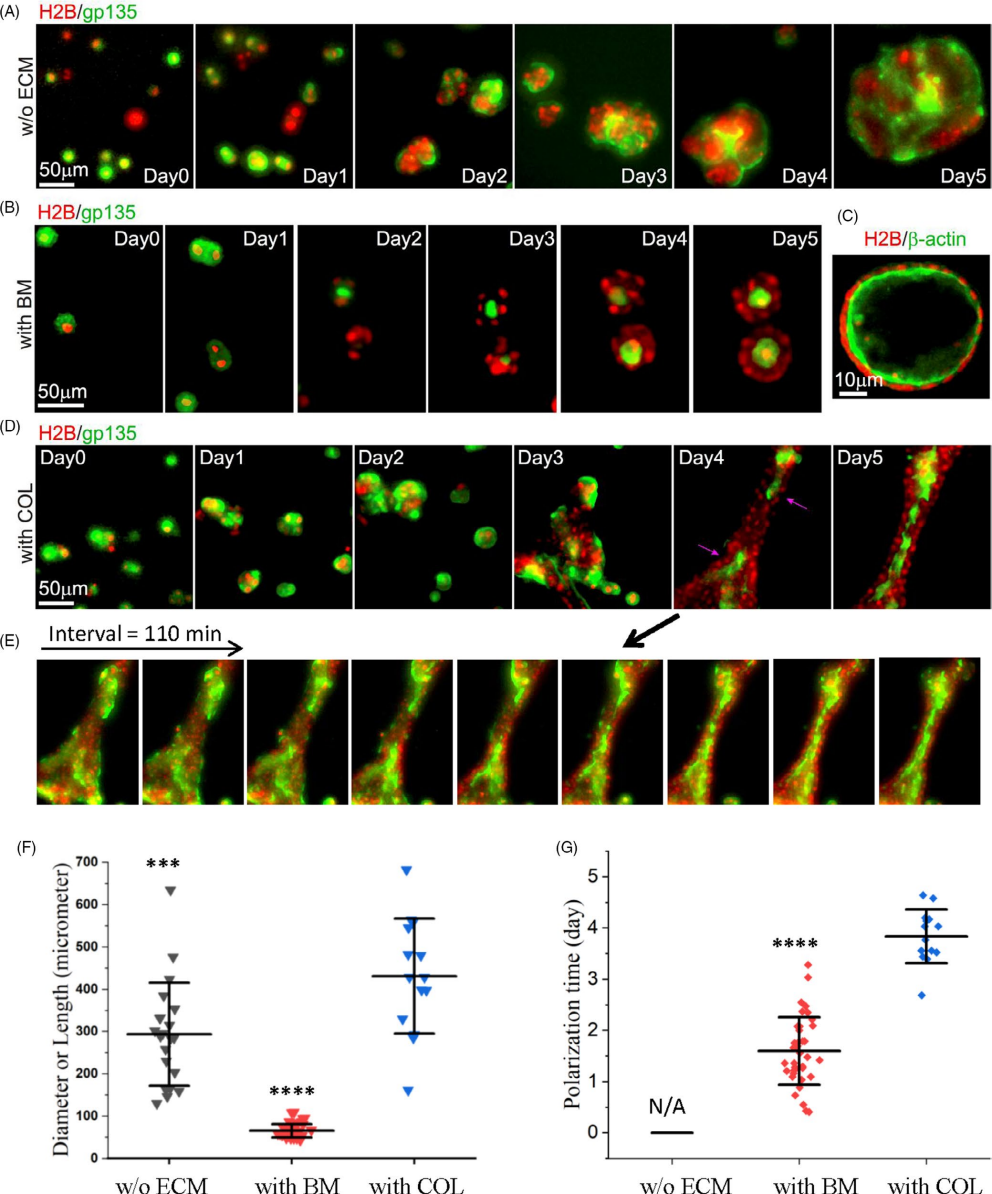
Distinct dynamics of cell coordination in response to soluble BM/COL. MDCK cells express H2B‐mCherry and GFP‐gp135 or β‐actin‐GFP to indicate cell nucleus and apicobasal polarity, respectively. Cells were cultured on BM gel with or without soluble ECM in the medium and positioned on the microscope by change with fresh medium every day. Time‐lapse fluorescence images were taken with 22 min interval time per cycle in the following days. (A and B) Represented time series of 3‐D projected epifluorescence images (gp135: green, H2B: red) for cells in medium (A) without ECM (n > 10), (B) with 2% basement membrane (BM) components (n > 10) (also seen in Movies 1&2, interval = 22 min). (C) Represented optical sectioning from scanning microscopy β‐actin: green, H2B: red) to demonstrate lumen formation with polarized actin distribution after culturing cells in medium containing 2% BM for 14 days. (D) Represented time series of 3‐D projected epifluorescence images for cells in medium with 20 µg/mL COL (also seen in Movie S3) (n = 14). The purple arrows indicate the polarization initiating from the local regions of the tubule. (E) The collective apicobasal polarization along the tubular axis (n = 6). The time‐sequence images (interval: 22 min*5 = 110 min) showed the spatial distribution of gp135 relative to cell nuclei (H2B) during the polarization. (F) Size quantification of the diameters (along the long axis) of unpolarized clusters without ECM (n = 21) and polarized lobular lumens with BM (n = 50), and the length of polarized tubes with COL (n = 14) around Day 5. (G) The timing quantification when lumens (n = 38) or tubules (n = 14) got polarized under culture with 2% BM or 20 µg/mL COL in the medium. N/A refers to ‘not applicable for polarization’ without ECM in the medium. The data quantification (mean ± SD) was performed by using ImageJ and Origin. *** and **** represent significant difference with *P* < 10^‐2^ and 10^‐6^ in comparison with the group ‘with COL’ from Student's *t* test analysis

We first examined cellular dynamics on BM gels in the presence or absence of soluble BM. Without soluble BM, time‐lapse microscopy revealed that individual cells proliferated into small clusters, which continuously grew and merged with each other without general polarity formation (through the 5‐day period of observation, Movie S1) (Figure 2A, Movie S1). By contrast, with soluble BM, most clusters stopped merging and became polarized on the 2nd‐3rd day (Figure 2B). Here, the timing of polarization was defined by the conversion of gp135 from the outer layers to the inner areas of clusters/cysts (Figure 2B, and Movie S2). The lumen cultured by MDCK cells (H2B‐mCherry/GFP‐β‐actin) displays actin ring at the apical side (Figure 2C), which was consistent with the previous report.^32^

We next examined the cellular dynamics on BM gels with soluble COL. Similar to the observation with soluble BM (Figure 2B), cells were found to form clusters which proliferated and coalesced. However, the coalescence did not stop on the 3rd day but instead clusters continued to fuse into a long‐range (>200 µm), tubule‐like structure (Figure 2D). By then, cells started to polarize through a collective conversion of gp135 from the outer layer to the inner area of tubule (Figure 2D, Movie S3). From the time‐sequence images, the polarization in the presence of soluble COL may proceed collectively along the axis of tubule in the same cluster in which the spatial localization of gp135 turned over from the boundary to the centre of the tubule along the polarization process (Figure 2E, Movie S3). The way of the tubular assembly had impact on whether there was typical collective polarization (~30%). During the coalescence, we often observed mutual attraction of clusters on the field of views under the time‐lapse microscopy (Movie S3). Such prolonged coalescence was not observed in the case where cells were surrounded with soluble BM, suggesting that it is a feature of COL‐cell interactions.

From the size quantification, unpolarized clusters (w/o ECM) grew into hundreds of micrometres in diameter, and polarized lobular lumens (with BM) were generally within 100 μm, while the length of the polarized tubes (with COL) reached ~ 400 μm at average (Figure 2F). The polarization time was much different between lobular lumens (generally within 2 days) and tubes (~4 days) (Figure 2G), which suggests different morphogenetic mechanism coordinated with BM and COL.

### 3.3 Cell polarization in the fluidic phase with ECM assembly

If the coordination of cell positioning and polarization requires the formation of ECM scaffolds, the results above suggest that it is the scaffold mediated by cell‐ECM interaction in the fluidic phase that determines epithelial morphology and coordinates polarity. To see how such scaffolds are formed and affect cell positioning and polarization, we performed immuno‐staining of laminin (a major component in BM^33^) and COL on polarized lobules and tubules.

**FIGURE 3:**
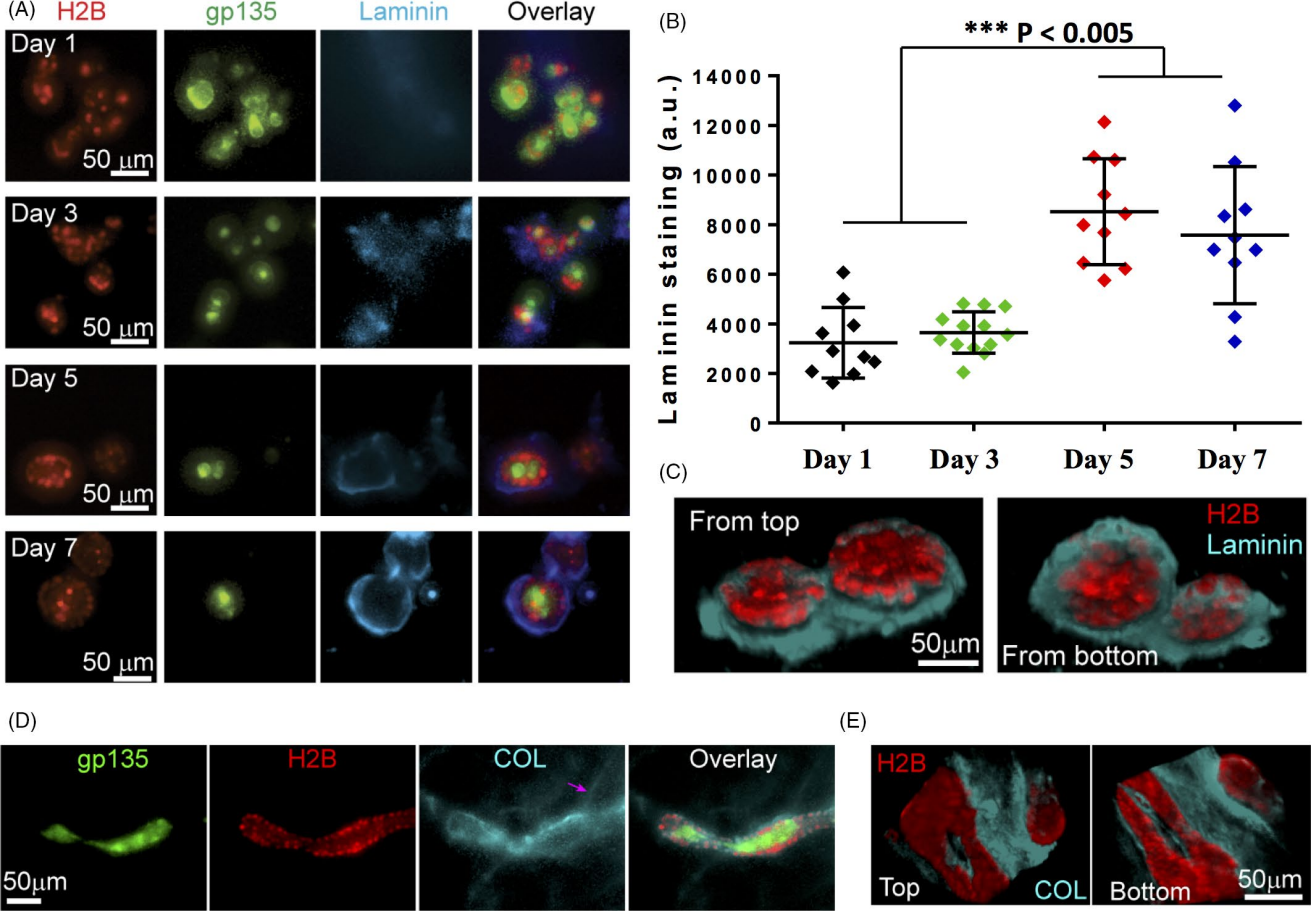
Cell polarization in the fluidic phase with and without direct cell‐ECM contact. (A, B**)** Lateral assembly of laminin around the luminal structure. MDCK cells were cultured for 1‐7 days on BM gels with 2% BM in the medium, followed by laminin immuno‐staining. (A) Represented fluorescent images of gp135‐GFP (green), H2B‐mCherry (red), laminin staining (cyan) and their overlay. (B) Fluorescence quantification of laminin staining on the cultured MDCK clusters (A). The graph data with individual dots (mean ± SD) show the average fluorescence intensity over the entire clusters with typical staining signals. a.u.: arbitrary unit. (C) Represented images of 3‐D reconstructed, top and bottom views of cell nucleus (red) and laminin (cyan) on the spherical lobules after 12‐day culture (also seen in Movie S4). (D) Represented fluorescent images of gp135‐GFP (green), H2B‐mCherry (red), COL staining (cyan) and their overlay of the tubules (n = 15). MDCK cells were cultured for 12 days on BM gels with COL (20 µg/mL) in the medium, followed by COL immuno‐staining. Note the formation of linear structure of COL (pink arrow). (E) Represented images of 3‐D reconstructed, top and bottom views of cell nucleus (red) and COL (cyan) on the tubule after 12‐day culture. For (C & E), images were taken by confocal scanning microscope and created by ImageJ 3‐D reconstructions. Note the lateral condensation of laminin (COL) around the lobules (tubule) (outlined by nucleus) and the absence of laminin (COL) assembly on the top and bottom

We first examined how laminin is distributed on cells seeded on BM gels with or without soluble BM. In the presence of soluble BM, condensed laminin was found at the outer layers of clusters after 3 days of culture (Figure 3A), and the density of laminin increased during the culture time (Figure 3B), indicating the laminin assembly along with the cluster growth. After 7 days, most clusters were found to form polarized lobules surrounded by dense laminin (Figure 3A). By contrast, laminin was not found at the outer layers of clusters in the absence of soluble BM (Figure S1), where clusters continuously grew and coalesced without polarity formation (Movie S1), suggesting that laminin assembled around clusters might block coalescence and induce polarization.

In the conventional model, the development of epithelial apicobasal polarity requires coordinated cell‐ECM interaction at the basal side^14^, ^34^ and cell‐cell interactions^2^, ^10^ at the lateral side of each individual cell. To ascertain whether this is the case in our in vitro assays where cell‐ECM interactions at the fluid phase appeared to play the deterministic role in apicobasal polarity formation, we examined the distribution of laminin around the clusters undergoing polarization. Cells on BM gels were cultured for 12 days with soluble BM to form polarized lobular lumens. Confocal scanning microscopy was then performed to construct 3‐D views of laminin and lumens with the lumens outlined by H2B‐mCherry signal from cell nucleus. Surprisingly, no complete, uniform assembly of laminin around the lobular lumens was found. Instead, assembled laminin was found to form a lateral platform at the lateral side of each lumen, rather than on the top (facing the medium) or bottom (facing the BM gel) (Figure 3C, and Movie S4), whereas cells at the entire lumen were polarized (Figure 1C). These results might also suggest that cells at the top of lumen could maintain polarity in the absence of direct cell‐ECM contact. The laminin platform appears as a 2‐D network compatible with the native topology of BM.^35^

To see whether cells can also polarize without direct cell‐ECM contact in the presence of soluble COL, we examined the distribution of COL around the clusters that underwent polarization on BM gels with soluble COL in the medium. After 12 days of culture, cells formed tubules and found associated with linear structures of COL (Figure 3D, pink arrow). Confocal scanning microscopy revealed that COL was condensed at the lateral sides, but not the top or bottom of tubules (Figure 3E), and similar to the results in the case with soluble BM (Figure 3C), cells at the entire tubules were polarized (Figure 3D). These suggest that those cells without ECM interaction might maintain their polarity through cell‐cell interactions in the lobules and tubes.

### 3.4 The expansion of dividing cells into fine spherical lobular structure under polarization

To further understand how cells maintained apicobasal polarity at the entire epithelial structures with only partial cell‐ECM interactions during the culture, we carried time‐lapse confocal imaging to observe the growth of dividing cells into polarized lobules. This experiment had certain technical challenges: due to the smaller field by confocal imaging than the wide‐field epi‐microscopy, the motile cell samples could move out of the views more likely; second, confocal imaging had higher photo‐toxicity during the point‐by‐point scanning and z‐stack imaging, which could cause damage on cell samples. To manage to get some time‐lapse samples successfully, we tried to lower the excitation laser power (25 mW) and used long interval time (3 or 4 hours).

**FIGURE 4:**
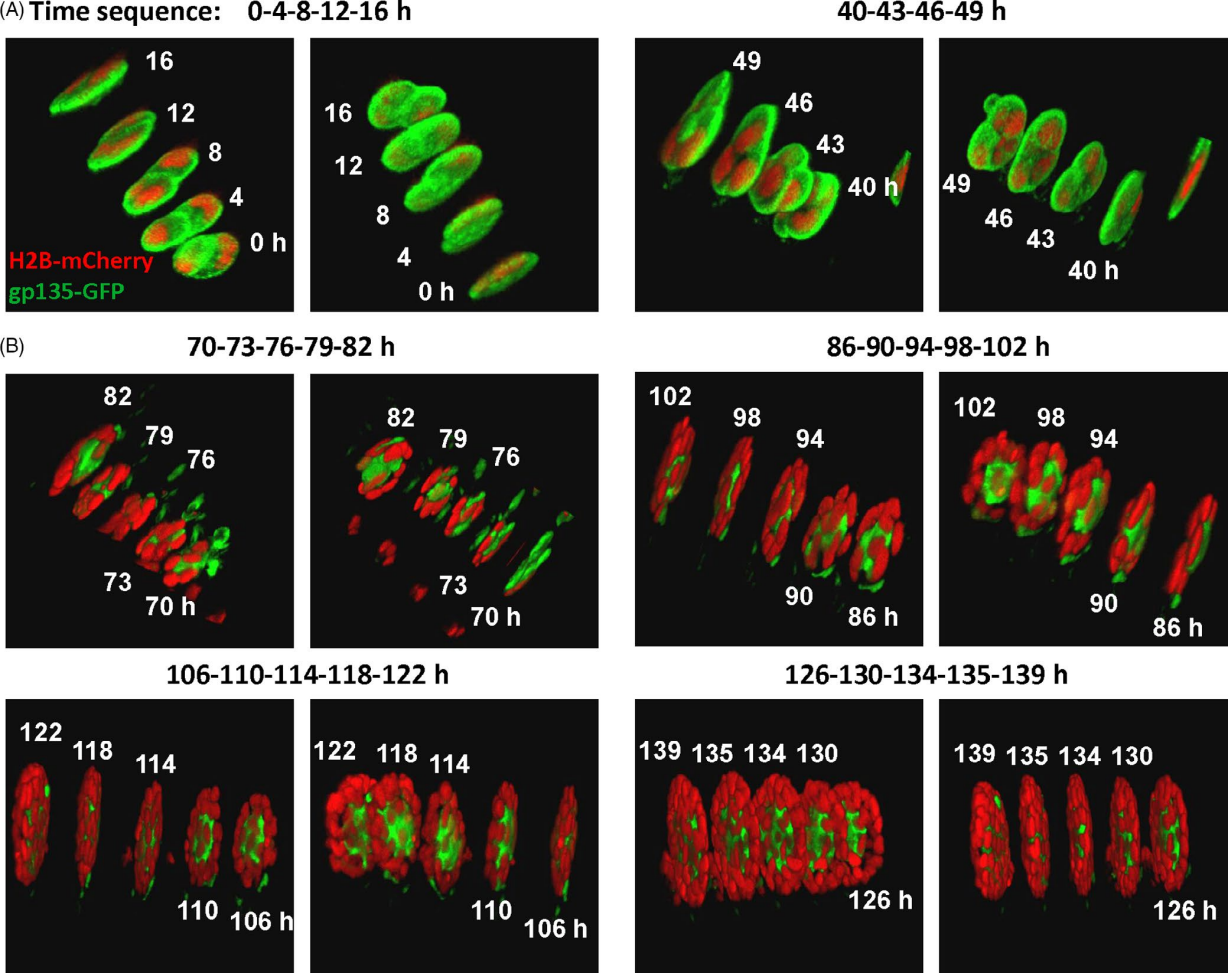
The expansion of dividing cells into polarized lobule. The MCDK cells expressing gp135‐GFP and H2B‐mCherry were seeded on BM gel with 2% BM in medium, and two‐photon confocal imaging started one day later. The wavelengths of excitation light were 890 nm for GFP and ~ 1100 nm for mCherry; the step size in z‐direction was 1.0 µm. Time‐lapse imaging was taken with the interval time of 3 or 4 h. Medium was changed every day along with focus/position corrections. Acquired confocal images were processed by two channels (GFP and mCherry) overlay and 3‐D reconstructions. Each image here shows the 3‐D view of 4 or 5 time‐points at the labelled time (in hours, and the starting time was set as zero), and two images for each time point were displayed from different angles (generally from the top and the bottom views). (A) The polarity distribution at early stage in the cluster with a few cells. (B) The polarity distribution during growth of the cluster into fine 3‐D lobular structure. More detailed 3‐D views are shown in Movie S5. A note: the images of (A, B) were acquired at the same position in one experiment, but may not be from the exact same sample during the re‐focusing process as cells were motile at the early stage

As the cell cluster growth shown in Figure 4 with more detailed views in Movie S5, the cell sample started at two‐cell stage, and continued to divide into 3‐ and 4‐cell cluster without polarization (located on one plane) on the next day (Figure 4A); late on the third day, the polarization was occurring in one cluster (not sure whether the same one from the beginning), and in the following days, the cluster continued to grow and expand into fine 3‐D lobular structure with maintained polarization, suggesting the possibility of cell divisions under polarized status in the lobule (Figure 4B). It is noted that the polarization of the cell cluster may occur in a collective way and gp135 was turned over into the cluster from one local side (Figure 4B, the first panel), which also indicates possible intercellular communications. These time‐sequence images indicate that the cell cluster got polarized at early stage, and grew into fine‐constructed 3‐D lobule under maintained polarization. The result may help explain how cell polarization occurred on the entire lobules although there was only partial laminin coverage on the basal side (Figure 3C). Similar mechanism may be extended to the observed tubular structure with partial COL coverage. In considering that this is a descriptive data with limited work, we did not have explanation why ECM components were not assembled on the top of the lobule, or dig out more insights at current stage.

### 3.5 Culture of lobular and tubular epithelial structures under suspension conditions

The results above suggest two distinct, ECM‐dependent processes to coordinate epithelial morphogenesis in the fluidic phase. The first is that cells recruit soluble BM components which are known to form branched networks,^35^ to create a closed‐end (ie, restricted) scaffold surrounding individual cluster. Such scaffold provides a physical barrier to block the coalescence of clusters and allow them to proliferate and polarize within the restricted space (Figure 3A). The second process is that cells recruit soluble COL, which is known to form linear, bundled fibres,^36^, ^37^ to create an open‐ended (ie, unrestricted) scaffold (Figure 3D). In contrast to the first one, scaffold formed by soluble COL allows clusters to continuously merge with one another through long‐range interactions in the fluidic/semi‐fluidic phase.

**FIGURE 5:**
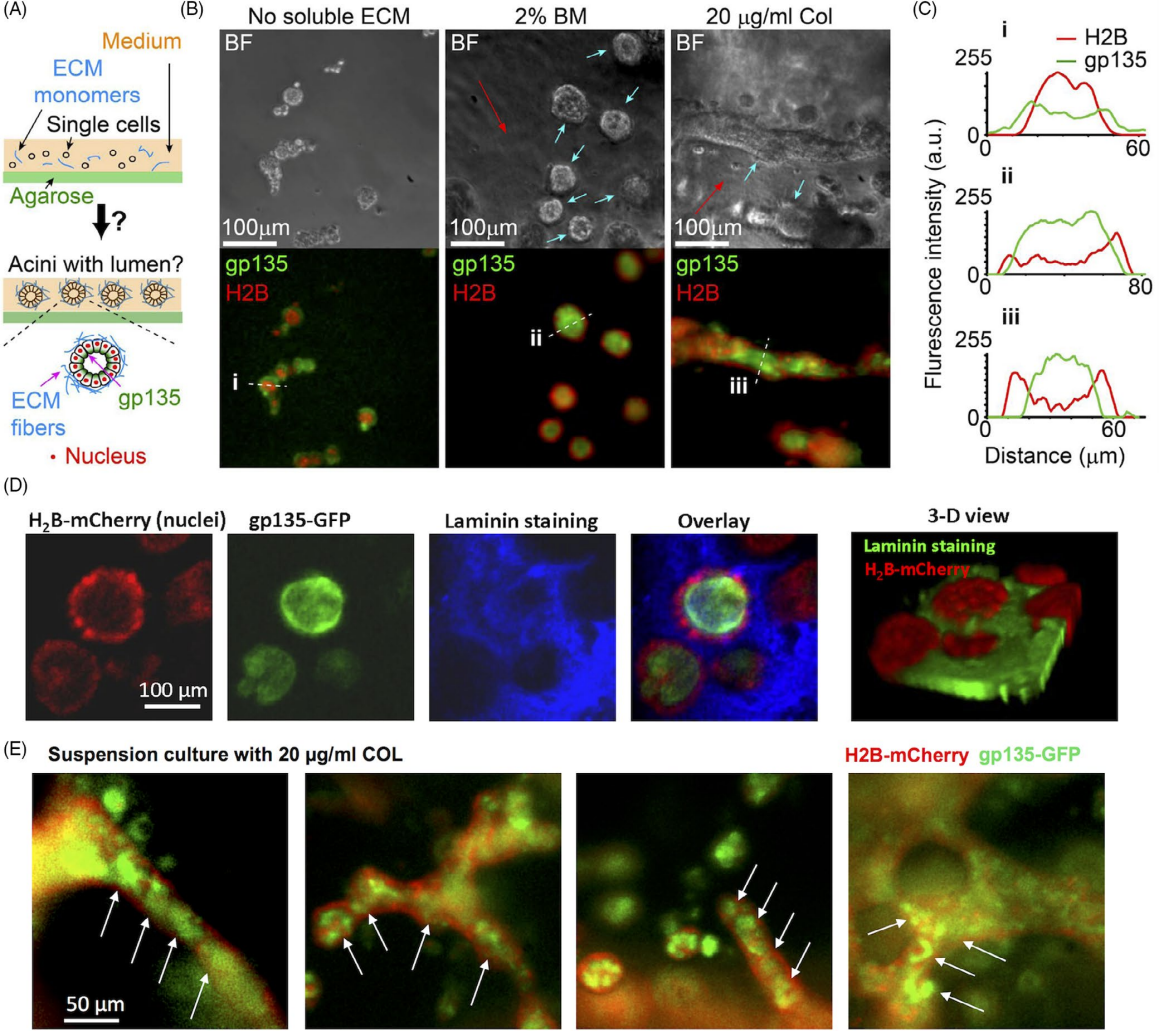
Cells form tubular and lobular structures above non‐adhesive substrates. (A) The design of suspension culture on agarose gel. Details are described in Methods. (B) Representative bright‐field and 3‐D projected epifluorescence images (Green: gp135, Red: H2B) for MDCK cells cultured in medium containing no ECM materials (Left), 2% BM (Middle), or 20 μg/mL COL components (Right) above 1% agarose gel for 12 days. Initial cell density: ~10^4^ cells/mL. To enhance visualization, out‐of‐focus signals were removed and only fluorescence signals from cells near the focal plane (indicated by blue arrows) were shown. The red arrows indicated the scaffolds self‐assembled from soluble BM or COL. (C) Fluorescence intensities of H2B‐mCherry and gp135‐GFP along the white dotted lines (from left to right) in the indicated H2B/gp135 overlay. The cell nucleus (red) located outside the apical marker (green) indicates polarity formation. (D) The 3‐D projected images of spherical lobules from suspension culture with 2% BM (~12 days). The laminin staining (blue) indicated the assembled scaffolds from soluble BM under suspension. The images were taken by confocal microscopy (20x objective). 3‐D projection of the images was obtained by collecting pixels with the maximal intensity through the entire z‐stack into a single plane, and the 3‐D view was constructed by the software ImageJ. (E) Formation of multi‐lumen polarity on the tubules under suspension culture. MDCK cells were cultured above agarose gel with 20 µg/mL COL in the medium for 12 days. The arrows indicate some of these small lumens on the tubules

These two processes were observed in the assays where cells were supported by pre‐assembled ECM, that is, a solid phase. To ascertain whether the solid phase is absolutely required in soluble ECM‐mediated epithelial cell polarization and morphogenesis, we repeated the assay in a suspension system where agarose gel was used to replace the solid phase and minimize cell‐substrate interaction (Figure 5A) (details seen in Methods). Cells (final concentration of ~ 1 × 10^4^ cells/mL) were mixed with medium containing no ECM (as the control) or soluble ECM components (2% BM or 20 µg/mL COL), spread in the suspension system, and examined for polarization after 7‐14 days.

Without soluble ECM components, cells in the suspension system were found to form clusters with a reverse polarity (ie, gp135 located at the outer boundaries of clusters, Figure 5B left panel), which is consistent with the previous finding.^38^ By contrast, cells mixed with soluble ECM components formed structures of polarized epithelium (Figure 5B, middle and right). Specifically, cells formed polarized lobular structures with BM (Figure 5B middle panel) and polarized tubular structures with COL (Figure 5B right panel), with the epithelial polarity indicated by the spatial distributions of H2B‐mCherry and gp135‐GFP signals (Figure 5C(i‐iii)). We noticed that under suspension culture with COL, separated small rooms (multi‐lumens) were formed in the tube during polarization (Figure 5B,E), instead of homogenous polarity through the tube on solid Matrigel (Figure 1B), which may reflect certain different ECM biomechanical microenvironments in the two culture systems. Specifically, the cells and ECM scaffolds floated in the medium during suspension culture, and the ECM scaffolds were also soft enough for antibody staining, whereas the ECM scaffold was bounded to glass surface during culture on the solid Matrigel which is also resistant to antibody staining.

Laminin immuno‐staining showed the distribution of ECM scaffold around the polarized lumen (Figure 5D), and laminin only covered the lateral sides of lobules, not at their top or bottom sides (Figure 5D (right)), which seemed similar to the results found in the lobules cultured on top of BM gels (Figure 3C). It was noted that soluble BM and COL in the medium could form scaffolds alone (Figure 5B and D), which assisted the formation of polarized epithelium. Thus, cell‐ECM interactions are required to initiate epithelial morphogenesis and create polarization at the tissue scale.

## 4 DISCUSSION

Emerging evidence suggests that cell‐ECM interactions are crucial in regulating tissue development, homeostasis and repair.^7^, ^39^, ^40^ Such interactions can occur on the solid phase (ie, the existing ECM scaffold) and in the fluidic phase where cells interact with soluble ECM components. Here, we showed the following findings based on the engineered MDCK cells stably co‐expressing H2B‐mCherry (nucleus) and gp135‐EGFP (apical marker): (1) the topology of native ECM components from cells‐mediated assembly helps define the morphology of epithelium tissue; (2) during tubulogenesis in the presence of soluble COL, the apicobasal polarization proceeds in a collective way along the axis of the tubule; (3) in the fluidic phase, cells can form apicobasal polarity throughout the entire lobule/tubule with ECM only partially covering the basal surfaces (ie, without ECM assembly at the top/bottom sides), and polarization occurred at early stage and was maintained through the entire lobular expansion; (4) under suspension culture with COL, the polarization was impaired by forming multi‐lumens on the tubules, suggesting the importance of ECM biomechanical microenvironment for tubulogenesis.

### 4.1 Self‐organization of polarized epithelium in the fluidic phase

First in our culture system, it is the scaffold formed by cell‐ECM interactions in the fluidic phase plays important roles in epithelial polarization and morphogenesis. This enables the self‐assembly of polarized epithelium. We note that 3‐D culture embedded in BM gels can form polarized lobules^9^ and has been used to study the polarization of cells obtained from tubular organs including the kidney, the liver and the prostate.^3^, ^41^, ^42^ Such 3‐D culture is different from our open‐system set‐up. In our set‐up, soluble ECM components secreted by cells are diluted, whereas in the 3‐D culture, they remain trapped within the space occupied by the cells. In turn, cell‐soluble ECM interactions are allowed in the 3‐D culture. Indeed, ECM components secreted from cells or degraded from gels are required for epithelial polarization in the 3‐D culture.^14^, ^43^ Consistently in the modified 3‐D culture (developed by Brugge et al.^19^) where cells are cultured on BM gels (mimicked by Matrigel in experiments), soluble BM is required for cells to form polarized lobules.^5^, ^12^, ^44^, ^45^ These observations highlight the criticalness of cell‐ECM interactions from the fluidic phase for epithelial polarization.

In general, soluble ECM can be assembled into scaffolds by ECM self‐assembly and/or by cell‐mediated nucleation process. The extent of cell‐soluble ECM interactions in scaffold assembly depends on the cell density and the soluble ECM concentration. In our open‐system set‐up, the concentration of soluble ECM is set low to eliminate or attenuate ECM self‐assembly. By contrast, the 3‐D model in previous studies^9^ used high concentrations of soluble ECM components to induce ECM self‐assembly.

It is likely that ECM scaffolds assembled by cell‐soluble ECM interactions possess a more physiological structure. For example, BM gels assembled in the absence of cells were found more resistant to laminin staining than those assembled by cells, as indicated by the difference of laminin staining (Figure 3A), which might result from difference of porosity. Indeed, previous reports showed that immune‐staining of cells in BM or COL gels requires a pre‐cleavage of gels by ECM proteinases.^14^, ^45^

### 4.2 Coordination of cell dynamics dependent on soluble ECM components

Our second finding is the distinct coordination of cell positioning and polarization in response to soluble BM (diluted Matrigel solution) and COL. With soluble COL, cell polarization was found to arise after the formation of elongated structures. By contrast, with soluble BM, cell polarization can occur without fusion of individual clusters. In addition, while cell polarization in soluble BM occurs almost simultaneously within the same cluster, cell polarization in soluble COL appears to follow a nucleation process in the cluster (Figure 2B and D, Movies 2&3). This collective polarization proceeded along the epithelial tubule (Figure 2E) might suggest existing intercellular signal communications at the cell‐population level during tubulogenesis. The selection of topology in the polarized epithelial clusters appears to correlate with the native topology of BM and COL (ie, lobular or linear/tubular).

It has been documented that cell‐ECM interactions through integrin signalling are crucial in regulating apicobasal polarity in epitheliums.^46^ By application of functional‐inhibitory antibodies, it was reported that integrin β1 is involved in the polarity regulation including the apical localization of gp135.^47^ Further studies of the downstream mechanism showed that integrin‐linking kinase (ILK) modulates laminin assembly and regulates integrin‐microtubule network for directional delivery of apical factors.^48^, ^49^ Multiple groups also revealed that small GTPases Rac and Rho signalling act downstream of integrins to mediate and maintain appropriate epithelial polarity.^16^, ^23^, ^50^, ^51^ Our work here added one piece of information to the scenario that biophysical factors like cells‐mediated assembly of ECM structure may play a role in directing the epithelial topology and polarity during morphogenesis. At this stage, nevertheless, we did not investigate how molecular signals from cell‐soluble BM/COL interactions lead to distinct dynamics in polarity formation.

### 4.3 Epithelial polarization with or without direct cell‐ECM contact

Our third finding is that cells can maintain polarity without a complete coverage of ECM scaffold around the lobular/tubular structures (Figures 3 and 5D), suggesting that some cells can be polarized without direct cell‐ECM interactions. Nevertheless, without ECM in the medium, cells were unable to form polarity (Figure 1 and Movie S1). Time‐sequence images from confocal microscopy indicate that cells are polarized in small cluster at early stage, and continue expanding into fine 3‐D lobular structure under maintained polarization (Figure 4), which reveals one mechanism of polarization on the entire lobules without complete ECM coverage. Previous studies reported that cadherins from cell‐cell junctional adhesions are important in maintaining epithelial structures and establishing epithelial cell polarity.^52^, ^53^, ^54^ It is a reasonable hypothesis that those cells without ECM coverage at the top of the lobules/tubules might maintain polarity under cell‐cell interactions or intercellular signalling communications, which need be confirmed from further study.

Under suspension culture with addition of soluble ECM, cells developed polarized lobules (with BM) and tubules (with COL) (Figure 5B), indicating cell‐ECM interactions in directing the self‐organization of epithelium. Interestingly, multiple lumens were formed on the tubes under suspension culture with COL (Figure 5E), instead of the homogenous polarity through the tube on solid BM gel (Figure 1B). This suggests that epithelial tubulogenesis is mediated by ECM microenvironment. Consistently from the previous report, perturbing cell mechanics by inhibition of ROCK‐Myosin II pathway also resulted in multiple lumens during tubulogenesis, due to impaired cell motility.^24^ The observations of multi‐lumen tubes support that the apicobasal polarization is also mediated in mechanical way beside chemical signals.

## 5 CONCLUSIONS

In summary, our results highlight the criticalness of cell‐ECM interactions in the fluidic phase for epithelial polarization and morphogenesis. Importantly, we show how a physiological environment can spontaneously emerge through cell‐soluble ECM interactions, and the native structure of self‐assembled ECM helps define the topology of self‐organized epithelium. The collective polarization during lobular/tubular morphogenesis may imply the existence of intercellular communications. In the fluidic phase, the polarization occurs in the small cluster at early stage and is maintained through the expansion into fine 3‐D epithelial structures. These findings may add a biophysical aspect on understanding how epithelial cells develop and coordinate their polarity and positioning at tissue scales in vivo, as well as in the engineering of artificial tissues in vitro.

## CONFLICT OF INTEREST

The authors declare no conflict of interest in this work.

## AUTHOR CONTRIBUTIONS

M. Ouyang and C. Guo designed the research and performed data analysis; M. Ouyang, J‐Y. Yu, C. Guo conducted the experiments; J‐Y. Yu, Y. Chen and C. Guo constructed the microscopes and developed the imaging programs; L. Deng provided discussion and certain fund support; M. Ouyang, L. Deng and C. Guo prepared the manuscript.

## Supporting information

Supplementary Material

Movie S1

Movie S2

Movie S3

Movie S4

Movie S5

## Data Availability

The data that support the findings of this study are available from the corresponding author upon reasonable request.

## References

[1] Quintin S , Gally C , Labouesse M . Epithelial morphogenesis in embryos: asymmetries, motors and brakes. Trends Genet. 2008;24:221‐230. 18375008 10.1016/j.tig.2008.02.005

[2] Bryant DM , Mostov KE . From cells to organs: building polarized tissue. Nat Rev Mol Cell Biol. 2008;9:887‐901. 18946477 10.1038/nrm2523 PMC2921794

[3] Martin‐Belmonte F , Mostov K . Regulation of cell polarity during epithelial morphogenesis. Curr Opin Cell Biol. 2008;20:227‐234. 18282696 10.1016/j.ceb.2008.01.001

[4] St Johnston D , Ahringer J . Cell polarity in eggs and epithelia: parallels and diversity. Cell. 2010;141:757‐774. 20510924 10.1016/j.cell.2010.05.011

[5] Bryant DM , Datta A , Rodriguez‐Fraticelli AE , Peranen J , Martin‐Belmonte F , Mostov KE . A molecular network for de novo generation of the apical surface and lumen. Nat Cell Biol. 2010;12:1035‐1045. 20890297 10.1038/ncb2106 PMC2975675

[6] Cait J , Hughes MR , Zeglinski MR , et al. Podocalyxin is required for maintaining blood‐brain barrier function during acute inflammation. Proc Natl Acad Sci U S A. 2019;116:4518‐4527. 30787191 10.1073/pnas.1814766116 PMC6410846

[7] Klinkert K , Rocancourt M , Houdusse A , Echard A . Rab35 GTPase couples cell division with initiation of epithelial apico‐basal polarity and lumen opening. Nat Commun. 2016;7:11166. 27040773 10.1038/ncomms11166 PMC4822036

[8] Mrozowska PS , Fukuda M . Regulation of podocalyxin trafficking by Rab small GTPases in 2D and 3D epithelial cell cultures. J Cell Biol. 2016;213:355‐369. 27138252 10.1083/jcb.201512024 PMC4862332

[9] Blaschke RJ , Howlett AR , Desprez PY , Petersen OW , Bissell MJ . Cell differentiation by extracellular matrix components. Methods Enzymol. 1994;245:535‐556. 7760750 10.1016/0076-6879(94)45027-7

[10] Dickinson DJ , Nelson WJ , Weis WI . A polarized epithelium organized by beta‐ and alpha‐catenin predates cadherin and metazoan origins. Science. 2011;331:1336‐1339. 21393547 10.1126/science.1199633 PMC3152298

[11] Mailleux AA , Overholtzer M , Brugge JS . Lumen formation during mammary epithelial morphogenesis: insights from in vitro and in vivo models. Cell Cycle. 2008;7:57‐62. 18196964 10.4161/cc.7.1.5150

[12] Martin‐Belmonte F , Gassama A , Datta A , et al. PTEN‐mediated apical segregation of phosphoinositides controls epithelial morphogenesis through Cdc42. Cell. 2007;128:383‐397. 17254974 10.1016/j.cell.2006.11.051 PMC1865103

[13] Nelson WJ . Adaptation of core mechanisms to generate cell polarity. Nature. 2003;422:766‐774. 12700771 10.1038/nature01602 PMC3373010

[14] O'Brien LE , Jou TS , Pollack AL , et al. Rac1 orientates epithelial apical polarity through effects on basolateral laminin assembly. Nat Cell Biol. 2001;3:831‐838. 11533663 10.1038/ncb0901-831

[15] Rozario T , DeSimone DW . The extracellular matrix in development and morphogenesis: a dynamic view. Dev Biol. 2010;341:126‐140. 19854168 10.1016/j.ydbio.2009.10.026 PMC2854274

[16] Yu W , Datta A , Leroy P , et al. Beta1‐integrin orients epithelial polarity via Rac1 and laminin. Mol Biol Cell. 2005;16:433‐445. 15574881 10.1091/mbc.E04-05-0435 PMC545874

[17] Dhimolea E , Maffini MV , Soto AM , Sonnenschein C . The role of collagen reorganization on mammary epithelial morphogenesis in a 3D culture model. Biomaterials. 2010;31:3622‐3630. 20149444 10.1016/j.biomaterials.2010.01.077

[18] Wozniak MA , Desai R , Solski PA , Der CJ , Keely PJ . ROCK‐generated contractility regulates breast epithelial cell differentiation in response to the physical properties of a three‐dimensional collagen matrix. J Cell Biol. 2003;163:583‐595. 14610060 10.1083/jcb.200305010 PMC2173660

[19] Muthuswamy SK , Li D , Lelievre S , Bissell MJ , Brugge JS . ErbB2, but not ErbB1, reinitiates proliferation and induces luminal repopulation in epithelial acini. Nat Cell Biol. 2001;3:785‐792. 11533657 10.1038/ncb0901-785 PMC2952547

[20] Paszek MJ , Zahir N , Johnson KR , et al. Tensional homeostasis and the malignant phenotype. Cancer Cell. 2005;8:241‐254. 16169468 10.1016/j.ccr.2005.08.010

[21] Guo CL , Ouyang M , Yu JY , Maslov J , Price A , Shen CY . Feature Article: From the Cover: Long‐range mechanical force enables self‐assembly of epithelial tubular patterns. Proc Natl Acad Sci U S A. 2012;109:5576‐5582. 22427356 10.1073/pnas.1114781109 PMC3326479

[22] Ekblom P , Alitalo K , Vaheri A , Timpl R , Saxen L . Induction of a basement membrane glycoprotein in embryonic kidney: possible role of laminin in morphogenesis. Proc Natl Acad Sci U S A. 1980;77:485‐489. 6987652 10.1073/pnas.77.1.485 PMC348296

[23] Bryant DM , Roignot J , Datta A , et al. A molecular switch for the orientation of epithelial cell polarization. Dev Cell. 2014;31:171‐187. 25307480 10.1016/j.devcel.2014.08.027 PMC4248238

[24] Kim M , Shewan AM , Ewald AJ , Werb Z , Mostov KE . p114RhoGEF governs cell motility and lumen formation during tubulogenesis through a ROCK‐myosin‐II pathway. J Cell Sci. 2015;128:4317‐4327. 26483385 10.1242/jcs.172361 PMC4712812

[25] Wang J , Guo J , Che B , Ouyang M , Deng L . Cell motion‐coordinated fibrillar assembly of soluble collagen I to promote MDCK cell branching formation. Biochem Biophys Res Commun. 2020;524:317‐324. 31996308 10.1016/j.bbrc.2020.01.019

[26] Meder D , Shevchenko A , Simons K , Fullekrug J . Gp135/podocalyxin and NHERF‐2 participate in the formation of a preapical domain during polarization of MDCK cells. J Cell Biol. 2005;168:303‐313. 15642748 10.1083/jcb.200407072 PMC2171597

[27] Ballestrem C , Wehrle‐Haller B , Imhof BA . Actin dynamics in living mammalian cells. J Cell Sci. 1998;111(Pt 12):1649‐1658. 9601095 10.1242/jcs.111.12.1649

[28] Sato Y , Poynter G , Huss D , et al. Dynamic analysis of vascular morphogenesis using transgenic quail embryos. PLoS One. 2010;5:e12674. 20856866 10.1371/journal.pone.0012674 PMC2939056

[29] Lee GY , Kenny PA , Lee EH , Bissell MJ . Three‐dimensional culture models of normal and malignant breast epithelial cells. Nat Methods. 2007;4:359‐365. 17396127 10.1038/nmeth1015 PMC2933182

[30] Ecay TW , Valentich JD . Basal lamina formation by epithelial cell lines correlates with laminin A chain synthesis and secretion. Exp Cell Res. 1992;203:32‐38. 1426050 10.1016/0014-4827(92)90036-8

[31] Parry G , Lee EY , Farson D , Koval M , Bissell MJ . Collagenous substrata regulate the nature and distribution of glycosaminoglycans produced by differentiated cultures of mouse mammary epithelial cells. Exp Cell Res. 1985;156:487‐499. 3917927 10.1016/0014-4827(85)90556-7

[32] Ivanov AI , Hopkins AM , Brown GT , et al. Myosin II regulates the shape of three‐dimensional intestinal epithelial cysts. J Cell Sci. 2008;121:1803‐1814. 18460584 10.1242/jcs.015842

[33] Kalluri R . Basement membranes: structure, assembly and role in tumour angiogenesis. Nat Rev Cancer. 2003;3:422‐433. 12778132 10.1038/nrc1094

[34] Mostov K , Brakeman P , Datta A . Formation of multicellular epithelial structures. Novartis Found Symp. 2005;269:193‐200; discussion 200–195, 223–130. 16355541

[35] Ingber DE . Mechanical control of tissue morphogenesis during embryological development. Int J Dev Biol. 2006;50:255‐266. 16479493 10.1387/ijdb.052044di

[36] Kadler KE , Hill A , Canty‐Laird EG . Collagen fibrillogenesis: fibronectin, integrins, and minor collagens as organizers and nucleators. Curr Opin Cell Biol. 2008;20:495‐501. 18640274 10.1016/j.ceb.2008.06.008 PMC2577133

[37] Starborg T , Lu Y , Meadows RS , Kadler KE , Holmes DF . Electron microscopy in cell‐matrix research. Methods. 2008;45:53‐64. 18442705 10.1016/j.ymeth.2008.01.004

[38] Wang AZ , Ojakian GK , Nelson WJ . Steps in the Morphogenesis of a Polarized Epithelium. 1. Uncoupling the Roles of Cell Cell and Cell Substratum Contact in Establishing Plasma‐Membrane Polarity in Multicellular Epithelial (Mdck) Cysts. J Cell Sci. 1990;95:137‐151. 2351699 10.1242/jcs.95.1.137

[39] Behonick DJ , Werb Z . A bit of give and take: the relationship between the extracellular matrix and the developing chondrocyte. Mech Dev. 2003;120:1327‐1336. 14623441 10.1016/j.mod.2003.05.002 PMC2775453

[40] Sequeira SJ , Larsen M , DeVine T . Extracellular matrix and growth factors in salivary gland development. Front Oral Biol. 2010;14:48‐77. 20428011 10.1159/000313707

[41] Tanimizu N , Miyajima A , Mostov KE . Liver progenitor cells develop cholangiocyte‐type epithelial polarity in three‐dimensional culture. Mol Biol Cell. 2007;18:1472‐1479. 17314404 10.1091/mbc.E06-09-0848 PMC1838984

[42] Webber MM , Bello D , Kleinman HK , Hoffman MP . Acinar differentiation by non‐malignant immortalized human prostatic epithelial cells and its loss by malignant cells. Carcinogenesis. 1997;18:1225‐1231. 9214606 10.1093/carcin/18.6.1225

[43] Davis GE , Koh W , Stratman AN . Mechanisms controlling human endothelial lumen formation and tube assembly in three‐dimensional extracellular matrices. Birth Defects Res C Embryo Today. 2007;81:270‐285. 18228260 10.1002/bdrc.20107

[44] Datta A , Bryant DM , Mostov KE . Molecular regulation of lumen morphogenesis. Curr Biol. 2011;21:R126‐136. 21300279 10.1016/j.cub.2010.12.003 PMC3771703

[45] Kim SH , Park S , Mostov K , Debnath J , Hunt CA . Computational investigation of epithelial cell dynamic phenotype in vitro. Theor Biol Med Model. 2009;6:8. 19476639 10.1186/1742-4682-6-8 PMC2696420

[46] Manninen A . Epithelial polarity–generating and integrating signals from the ECM with integrins. Exp Cell Res. 2015;334:337‐349. 25597426 10.1016/j.yexcr.2015.01.003

[47] Ojakian GK , Schwimmer R . Regulation of epithelial cell surface polarity reversal by beta 1 integrins. J Cell Sci. 1994;107(Pt 3):561‐576. 7516342

[48] Akhtar N , Streuli CH . An integrin‐ILK‐microtubule network orients cell polarity and lumen formation in glandular epithelium. Nat Cell Biol. 2013;15:17‐27. 23263281 10.1038/ncb2646 PMC3701152

[49] Rudkouskaya A , Welch I , Dagnino L . ILK modulates epithelial polarity and matrix formation in hair follicles. Mol Biol Cell. 2014;25:620‐632. 24371086 10.1091/mbc.E13-08-0499 PMC3937088

[50] Li R , Pendergast AM . Arg kinase regulates epithelial cell polarity by targeting beta1‐integrin and small GTPase pathways. Curr Biol. 2011;21:1534‐1542. 21906945 10.1016/j.cub.2011.08.023 PMC3189484

[51] Yu W , Shewan AM , Brakeman P , et al. Involvement of RhoA, ROCK I and myosin II in inverted orientation of epithelial polarity. EMBO Rep. 2008;9:923‐929. 18660750 10.1038/embor.2008.135 PMC2529350

[52] Harris TJ , Peifer M . Adherens junction‐dependent and ‐independent steps in the establishment of epithelial cell polarity in Drosophila. J Cell Biol. 2004;167:135‐147. 15479740 10.1083/jcb.200406024 PMC2172516

[53] Jiang MC , Liao CF , Tai CC . CAS/CSE 1 stimulates E‐cadhrin‐dependent cell polarity in HT‐29 human colon epithelial cells. Biochem Biophys Res Commun. 2002;294:900‐905. 12061792 10.1016/S0006-291X(02)00551-X

[54] Koumarianou P , Gomez‐Lopez G , Santisteban P . Pax8 controls thyroid follicular polarity through cadherin‐16. J Cell Sci. 2017;130:219‐231. 27780871 10.1242/jcs.184291 PMC5394772

